# Application of Elastic networks and Bayesian networks to explore influencing factors associated with arthritis in middle-aged and older adults in the Chinese community

**DOI:** 10.3389/fpubh.2025.1437213

**Published:** 2025-04-09

**Authors:** Tao Zhong, Tianlun Li, Jiapei Hu, Jiayi Hu, Li Jin, Yuxuan Xie, Bin Ma, Dailun Hu

**Affiliations:** ^1^School of Public Health, Hebei Medical University, Shijiazhuang, China; ^2^Graduate School, The Second Affiliated Hospital of Hebei Medical University, Shijiazhuang, China; ^3^Graduate School, The First Affiliated Hospital of Soochow University, Suzhou, China; ^4^School of Medical, Molecular and Forensic Sciences, Murdoch University, Murdoch, WA, Australia; ^5^Centre for Healthy Ageing, Health Futures Institute, Murdoch University, Murdoch, WA, Australia; ^6^Department of Pathogenic Biology, Hebei Medical University, Shijiazhuang, China

**Keywords:** Bayesian networks, Elastic networks, arthritis, prediction, influencing factors

## Abstract

Bayesian networks (BNs) are an excellent machine learning algorithm for extensively exploring the influencing factors associated with many diseases. However, few researchers have used BNs to examine the influencing factors associated with arthritis in older adults in the Chinese community. Our aim has been to use BNs to construct a complex network of relationships between arthritis and its related influencing factors and to predict arthritis through Bayesian inference, thereby providing scientific references for its control and prevention. Data were downloaded from the 2015 China Health and Retirement Longitudinal Study (CHARLS) online database, a longitudinal survey of the middle-aged and older adults in China. Twenty-two variables such as smoking, depressive symptoms, age, and joint pain were included in this study. First, Elastic networks (ENs) were used to screen for features closely associated with arthritis, and we subsequently incorporated these features into the construction of the BNs model. We performed structural learning of the BNs based on the taboo algorithm and used the maximum likelihood method for parameter learning of the BNs. In total, 15,764 participants were enrolled in this study, which included 5,076 patients with arthritis. ENs identified 13 factors strongly associated with arthritis. The BNs consisted of 14 nodes and 24 directed edges. Among them, depressive symptoms and age were direct influences on arthritis, whereas gender was an indirect influence on the diseases. BNs graphically visualized the complex network of relationships between arthritis and its influences and predicted the development of arthritis through Bayesian inference. These results were in line with clinical practice. BNs thus have a wide range of application prospects.

## 1 Introduction

Arthritis is a common chronic disease characterized by joint pain, joint swelling, and joint dysfunction, with the common clinical types being rheumatoid arthritis, and osteoarthritis. This disease has become a critical public health problem worldwide, causing enormous psychological stress to patients and a huge economic burden to society (1). According to the 2019 Global Burden of Disease (GBD), more than 16.5 million people worldwide have rheumatoid arthritis, and about 190 million people are osteoarthritic (2, 3). According to statistics, about 31.4% middle-aged and older adults in China suffer from arthritis, the prevalence increasing with age (4). In this social environment, the exploration of risk factors for arthritis is important and beneficial for arthritis prevention.

Previous studies have reported arthritis with various risk factors such as age, gender, education, and cardiovascular disease (4, 5). However, these investigations have some limitations. They mainly rely on logistic regression based on independence to estimate the prevalence of arthritis and assess the strength of the association between various risk factors and arthritis by the odds ratio (OR), which does not allow the identification of direct or indirect risk factors. Bayesian networks (BNs), an artificial intelligence learning method that does not rely on rigorous statistical hypothesis testing (6), provide better solutions for exploring the complex relationships between diseases and their risk factors. BNs construct directed acyclic graphs to show potential relationships between multiple factors and use conditional probability tables to represent the strength of the association and to reason the probability of unknown nodes via the probability of known nodes. BNs demonstrate the connections between factors in a network-like structure that enables a more intuitive demonstration of the interactions between different factors.

BNs have many advantages over traditional models, including its ability to infer the probability of unknown nodes and its flexibility regarding the demonstration of the impact of relevant risk factors on arthritis. Of course, many factors are associated with the development of arthritis, and it is clearly inappropriate to include all of them in the BNs. Hence, the screening of variables becomes important. The Elastic networks (ENs), which combine the advantages of Lasso regression (7) and Ridge regression (8), are flexible and effective methods commonly used for variable screening (machine learning-based regression methods). We can now select factors closely associated with arthritis based on the EN and can explore the complex relationship between arthritis and these factors by using the BNs. However, to the best of our knowledge, no study has comprehensively explored the risk factors for arthritis in middle-aged and older adults in the Chinese community via BNs. We believe that our study will thus provide new insights into clinical practice, reduce the prevalence of arthritis in the middle-aged and older adult population, and provide information and recommendations for the prevention of arthritis and for interventions in the development of this disease. This study is therefore of great public health significance.

## 2 Materials and methods

### 2.1 Study design and data sources

The China Health and Retirement Longitudinal Study (CHARLS) aims to collect a set of high-quality microdata representing households and individuals of middle-aged and older adults aged 45 years and above in China to enable the analysis of the problems of the aging population in the country and to promote interdisciplinary research on aging issues. The national baseline survey was launched in 2011 and is now in its fifth edition. The latest edition of the national baseline survey of middle-aged and older adults is the fifth edition, which appeared in 2020 (9). The CHARLS baseline survey covers 150 districts and 450 villages/urban communities across the country, involving 17,708 people in 10,257 households, reflecting the overall situation of China's middle-aged and old population and ensuring a representative sample.

We used CHARLS Phase 3 data and examined data obtained from the CHARLS 2015 survey in this cross-sectional study. The data included demographic background, health status and functioning, biomarkers, and individual income. Inclusion criteria for this study were (1) individuals ≥45 years of age, and (2) no missing data information as to whether they had arthritis. A total of 15,764 subjects were included. Participants were selected for screening in a random manner, maintaining a 7:3 ratio between the training and validation groups. In total, 10,510 (70%) and 5,254 (30%) were assigned to the training and validation groups, respectively. Subjects were classified as having or not having arthritis.

### 2.2 Variables

Age was categorized as 45–55 years, 55–65 years, 65–75 years, or >75 years, sex as male and female, residence as urban or rural, educational level as primary school, middle school, or university, marital status as married, divorced, widowed, or never married, self-reported health status as very poor, poor, fair, good, or very good, sleep duration as ≤ 6 h, 6–7 h, 7–8 h, or ≥8 h, smoking and alcohol consumption as “yes” or “no”, life satisfaction as completely satisfied, very satisfied, somewhat satisfied, not very satisfied, or not at all satisfied, and assets as ≤ 3,000 Yuan, 3,000–8,000 Yuan, or ≥8,000 Yuan. The location of joint pain was defined as the answer to the question “In which part of the body do you feel pain? Please list all parts of the body where you currently feel pain”. The site of joint pain was categorized as “ < 2” or “≥2”. Body mass index (BMI) was calculated based on the participant's height and weight and was categorized as “ < 30 kg/m^2^” or “≥30 kg/m^2^”.

The assessment of daily living skills consisted of Basic Activities of Daily Living (BADL) and Instrumental Activities of Daily Living (IADL). BADL included six parameters: dressing, bathing or showering, eating, getting into or out of bed, using the toilet, and controlling urination and defecation. IADL included six parameters: doing household chores, preparing hot meals, shopping for groceries, making phone calls and taking medications, and managing money. Failure to complete any of the indicators was defined as either BADL or IADL impairment (10).

Depressive symptoms were assessed by the 10-item Center for Epidemiological Studies of Depression Scale (CESD-10), with a total depression score ranging from 0 to 30. Patients with a score of more than 9 were considered to have depressive symptoms (11, 12).

The CHARLS database collects information on respondents' doctor-diagnosed chronic diseases by asking “Have you been diagnosed by a doctor with any of the following diseases?”. Chronic diseases here included 14 diseases: high blood pressure, dyslipidemia, diabetes, cancer, chronic lung disease, liver disease, heart disease, stroke, kidney disease, stomach or digestive disease, emotional or mental problems, memory-related diseases, arthritis or rheumatism, and asthma. In this study, the presence of arthritis was defined based on the answer to the question, “Have you been diagnosed with arthritis or rheumatism by a doctor?”. The presence of physical pain, high blood pressure, dyslipidemia and diabetes, and heart disease were defined by “yes” or “no”. (5).

Some variables have missing values. First, we remove the features with more than 40% of missing values directly and then use the Random Forest (RF) method (13) (a machine learning method) of the “mice” package in R software to perform multiple interpolation.

### 2.3 Elastic networks

When a high degree of covariance or a high degree of two-by-two correlation occurs, Lasso regression does not take into account the correlation between the features and may force the removal of a predictive feature. Similarly, Ridge regression is unable to create models with zero coefficients for certain features making it unsuitable for feature selection. In 2005, Zou and Hastie proposed the ENs model, which is a convex combination of Lasso regression and Ridge regression (14). The equation for the ENs is as follows:


(1) $$\begin{array}{l} \begin{array}{c} \overset{\;\wedge }{\;B}=\arg_{\beta^{2}}^{\min}∥Y-X\beta {∥}_{2}^{2}+\lambda \left(\alpha ∥B∥1+\frac{\left(1-\;\alpha \right)}{2}∥B∥2\right) \end{array} \end{array}$$

where, λ represents the penalty coefficient, and β is the regression coefficient. The parameter α is a value from 0 to 1 and is used to adjust the penalty with λ. When α = 1, the EN is equivalent to Lasso regression; when α = 0, the EN is equivalent to Ridge regression. The EN combines the advantages of Lasso regression and Ridge regression, both in feature selection among a large number of redundant features and in handling highly correlated features.

### 2.4 Bayesian networks

Judea Pearl proposed a probabilistic graphical model for uncertainty reasoning, namely BNs in 1988 (15). BNs consist of a directed acyclic graph (DAG) and a conditional probability table (CPT) showing the degree of probabilistic reliance between factors. The DAG consists of nodes and directed edges, where each node represents a variable feature in the network. If variable X points to variable Y, then a direct probabilistic reliance exists between X and Y. In addition, if the new variable Z points to Y through X, then an indirect probabilistic reliance exists between Z and Y. CPT provides a quantitative description of the strength of the probabilistic reliance. The formula for the joint probability distribution on the function of all nodes in BNs is shown below:


(2) $$\begin{array}{l} P\left(x_{1},x_{2},\cdots \;,x_{n}\right)=P\left(x_{1}\right)P\left(x_{2}|x_{1}\right)\cdots P\left(x_{n}|x_{1},x_{2},\cdots \;,x_{n-1}\right)\\ \;=\Pi_{1}^{n}P\left(x_{i}|\pi \left(x_{i}\right)\right) \end{array}$$

where, π(x_i_) is the set of parent nodes of x_i_; when the value of π(x_i_) is known, x_i_ is conditionally independent of the other variables in (x_1_, x_2_,..., x_i − 1_).

### 2.5 Statistical analysis

Quantitative data are described using the mean plus the standard deviation, and qualitative data are described using percentages. We used *t*-tests and Chi-square tests to compare quantitative and qualitative data, respectively. The ENs were implemented based on the “Glmnet” package in R studio (4.2.3). The structure learning of BNs was implemented based on the forbidden function algorithm in the “bnlearn” package in R studio (4.2.3). The maximum likelihood estimation was used for the parameter learning of BNs. Bayesian inference and conditional probability distribution tables were obtained using Netica software. *p* < 0.05 was considered statistically significant.

## 3 Results

### 3.1 Characteristics of study population

Based on the inclusion criteria of not < 45 years of age, no missing outcome information and complete general information, 15,764 participants were included in this study. As shown in Table 1, a total of 5,076 participants had arthritis, of which 2,110 (41.8%) were male and 2,966 (58.4%) were female. The distribution of educational level was 4,847 (95.5%) had attended primary school, 220 (4.3%) middle school, and 8 (0.2%) university. The average age was 72 years.

**Table 1:** Baseline characteristics of participants.

	**Arthritis**	**No arthritis**	***p*-value**
*n*	5,076	10,688	
BADL = yes (%)	4,006 (79.1)	7,695 (72.1)	< 0.001
Depression [mean (SD)]	9.81 (6.92)	7.06 (5.92)	< 0.001
Drinking alcohol = no (%)	3,455 (68.1)	6,760 (63.3)	< 0.001
Educational level (%)			< 0.001
Primary school	4,847 (95.5)	9,815 (91.8)	
Middle school	220 (4.3)	806 (7.5)	
College	8 (0.2)	66 (0.6)	
IADL = yes (%)	1,433 (28.4)	1,728 (16.2)	< 0.001
Individual income (%)			< 0.001
< 3,000 Yuan	196 (27.5)	358 (13.8)	
3,000–8,000 Yuan	117 (16.4)	312 (12.0)	
>8,000 Yuan	400 (56.1)	1,924 (74.2)	
Marital status (%)			< 0.001
Married	4,272 (84.2)	9,516 (89.0)	
Divorced	26 (0.5)	102 (1.0)	
Widowed	734 (14.5)	989 (9.3)	
Never married	44 (0.9)	81 (0.8)	
Hypertension = no (%)	3,630 (71.5)	8,895 (83.2)	< 0.001
Dyslipidemia = no (%)	4,493 (88.5)	9,892 (92.6)	< 0.001
Diabetes = no (%)	4,713 (92.8)	10,249 (95.9)	< 0.001
Heart disease = no (%)	4,199 (82.7)	9,879 (92.4)	< 0.001
Arthritis = no (%)	0 (0.0)	10,688 (100.0)	< 0.001
Sex = female (%)	2,966 (58.4)	5,438 (50.9)	< 0.001
Smoke = no (%)	3,102 (61.1)	4,689 (56.8)	< 0.001
Self-report (%)			< 0.001
Very good	159 (6.2)	773 (14.7)	
Good	204 (8.0)	702 (13.3)	
Fair	1,240 (48.6)	2,698 (51.1)	
Poor	714 (28.0)	884 (16.8)	
Very poor	232 (9.1)	219 (4.2)	
Body pain = no (%)	2,727 (54.9)	8,032 (76.8)	< 0.001
Sleep duration [mean (SD)]	6.08 (2.11)	6.54 (1.82)	< 0.001
Life satisfaction (%)			< 0.001
Completely satisfied	282 (5.7)	732 (7.0)	
Very satisfied	1,573 (32.0)	4,017 (38.6)	
Somewhat satisfied	2,524 (51.4)	4,900 (47.1)	
Not very satisfied	403 (8.2)	628 (6.0)	
Not at all satisfied	128 (2.6)	133 (1.3)	
BMI [mean (SD)]	23.89 (4.08)	23.90 (3.80)	0.918
Age [mean (SD)]	71.73 (9.18)	67.44 (10.55)	< 0.001
Joint pain < 2 (%)	3,058 (60.2)	8,748 (81.8)	< 0.001
Residence = village (%)	4,093 (80.9)	7,564 (72.9)	< 0.001

In total, 10,688 people without arthritis were included in the study, with a mean age of 67 years. There were slightly fewer males (5,250, 49.1%) than females (5,438, 50.9%). In terms of educational level, more had a university education, and fewer had only a primary school education compared with the arthritis group. Detailed information on other variables can be found in Table 1.

### 3.2 Elastic networks regression

**Figure 1:**
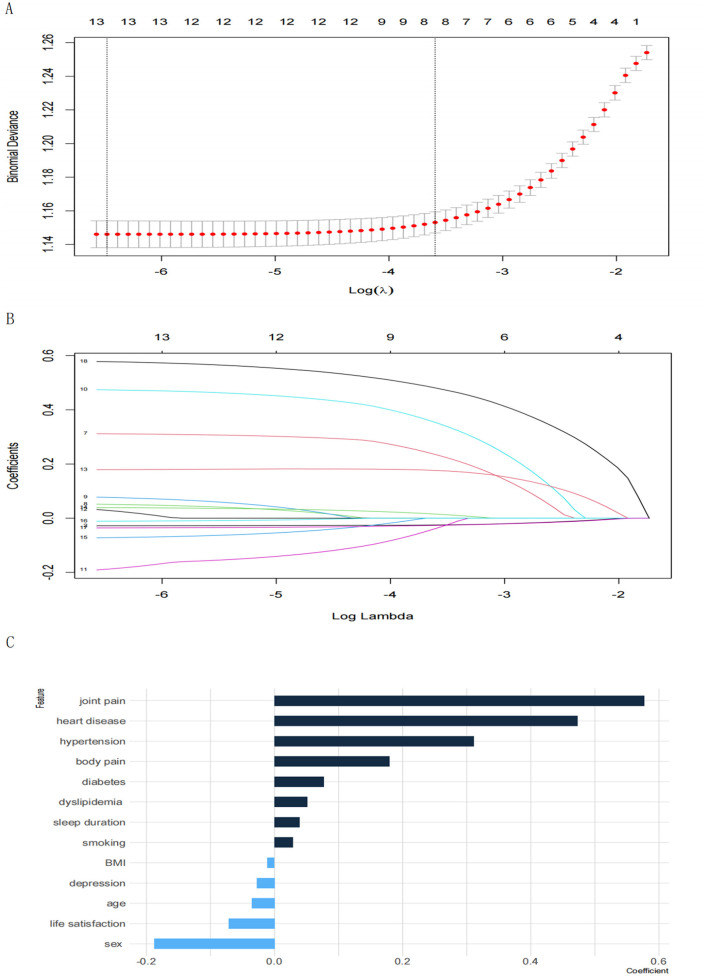
**(A)** Plot of lambda tuning for 10-fold cross-validation. The horizontal axis represents the logarithmic value of the penalty parameter λ in the regularization of the ENs (Log(λ)); the vertical axis represents Binomial Deviance, which is an error metric used to measure the performance of the model in cross-validation, where the smaller the deviation, the better the model performs. The leftmost dashed line corresponds to the Minimum Deviation point (min λ), the value of λ that minimizes the model error, when the model has the best predictive performance. **(B)** Coefficient trajectory plot. The horizontal axis represents the logarithmic value of the ENs penalty parameter λ (Log(λ)); the vertical axis represents the coefficient values for different features, with different lines indicating different features; **(C)** characteristic coefficient bar graph.

In this study, the proportion of missing values for individual income features and individual-reported health status features exceeded 40% and were removed outright. We used ENs to carry out the selection of the remaining features and optimized the key parameters to achieve optimal model efficacy through 10-fold cross-validation (α = 0.6, λ = 0.001535792). We compressed the coefficients of factors less closely related to arthritis to 0 and eliminated them. As shown in Figure 1, 13 factors were strongly associated with arthritis. These factors included depression score (−0.02778762), hypertension (0.31139245), dyslipidemia (0.05123738), gender (−0.18813527), age (−0.03560959), smoking (0.02900539), sleep duration (0.03879666) and body mass index (−0.01110007), joint pain (0.57742106), diabetes (0.07672528) and heart disease (0.47334178), life satisfaction (−0.07177999), and body pain (0.17915726). In ENs regression, the positive and negative values of the coefficients reflect the direction of the relationship between the predictor and target variables. A positive coefficient indicates that the predictor variable is positively associated with arthritis risk, whereas the opposite is a negative association with arthritis risk. This approach simplified the structure of the BNs by focusing on factors that were strongly associated with the development of arthritis.

### 3.3 Bayesian networks

**Figure 2:**
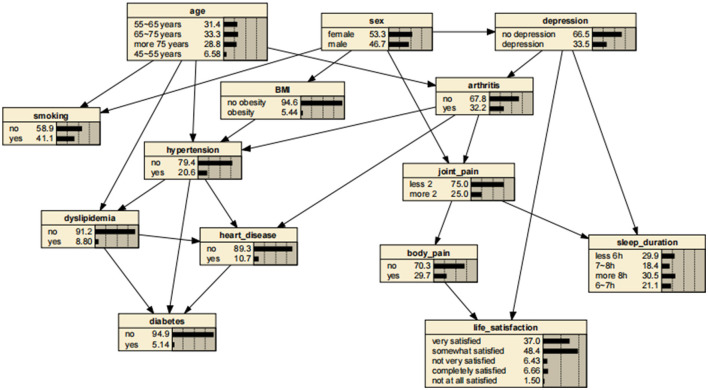
Bayesian networks for arthritis.

The BN structure included 14 variables and consisted of 14 nodes and 24 directed edges. Each node represented a variable, and a directed edge represented the existence of a probabilistic reliance between two variables. The numbers in Figure 2 represent the prior probability of each variable. In this case, the *a priori* probability of arthritis is 32.2% or P(arthritis) = 0.322.

BNs showed that age and depressive symptoms were parent nodes for arthritis, suggesting that these factors are directly related to the development of arthritis. In addition, gender, as a parent node for depressive symptoms, might have influenced the development of arthritis through depressive symptoms. In contrast, arthritis as a parent node for hypertension, heart disease, and joint pain might have directly contributed to the development of these diseases and symptoms.

**Figure 3:**
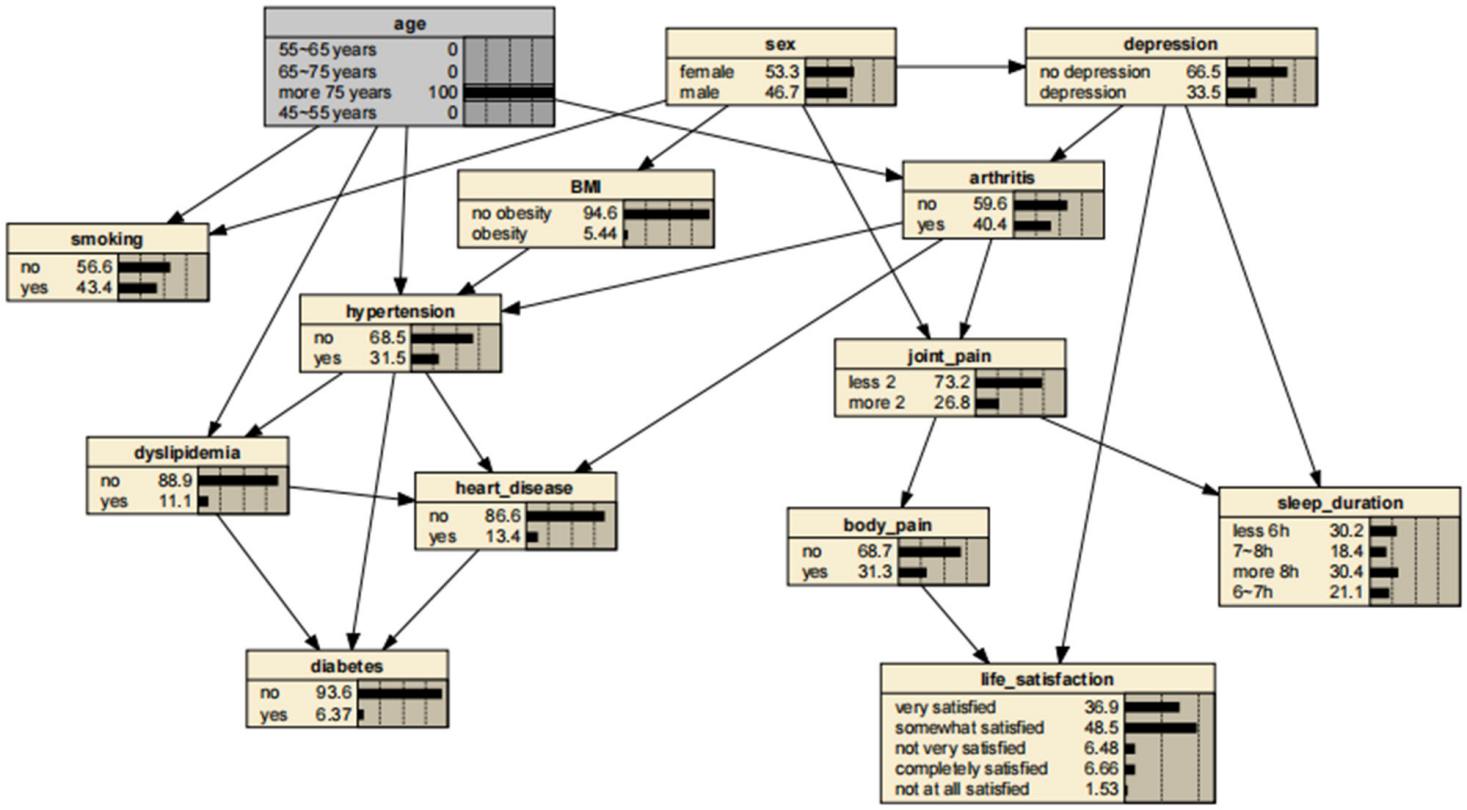
Bayesian networks for arthritis for those aged 75 years.

**Figure 4:**
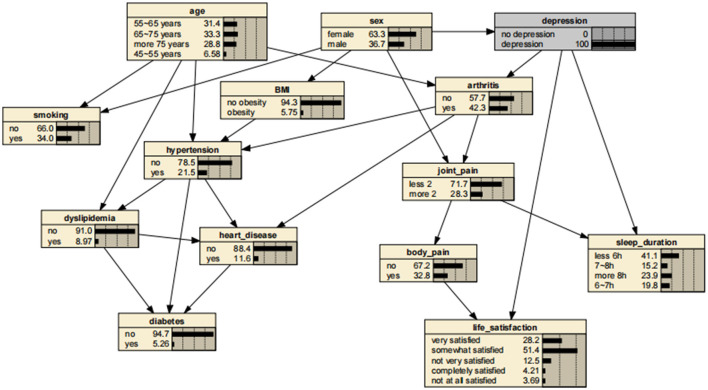
Bayesian networks for arthritis for those who have depressive symptoms.

**Figure 5:**
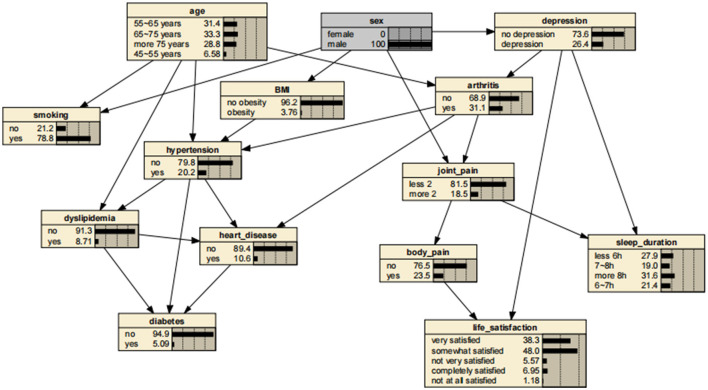
Bayesian networks for male.

**Figure 6:**
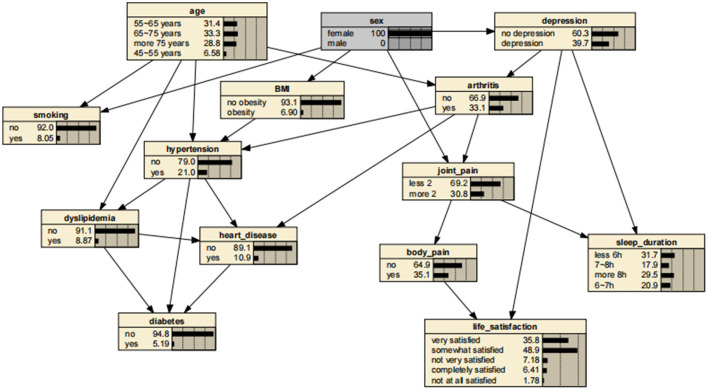
Bayesian networks for female.

**Figure 7:**
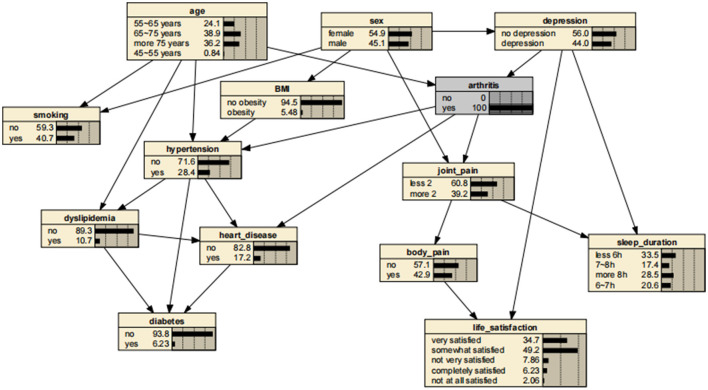
Bayesian networks for those who have arthritis.

A significant advantage of the BNs is that it can analyze the effect of various factors on the outcome by calculating the conditional probability P(y|x_i_), inferring the probability of unknown nodes based on the probability of known nodes and predicting the likelihood of the arthritis. Thus, the developed BNs can independently predict the risk of arthritis in middle-aged and older adults in the Chinese community. The prevalence of arthritis increased from the previous rate of 0.322 to 0.404, if a person was older than 75 years, as shown in Figure 3. In addition, depressive symptoms can promote arthritis, increasing the risk 1.31-fold to 0.423. The prevalence rate for women was 0.331, compared with 0.311 for men, suggesting that women are more likely to suffer from arthritis than are men. Arthritis also promotes the development of cardiovascular disease, suggesting a link between arthritis and cardiovascular disease. These findings are shown in Figures 4–7.

### 3.4 Prediction of BN model

**Figure 8:**
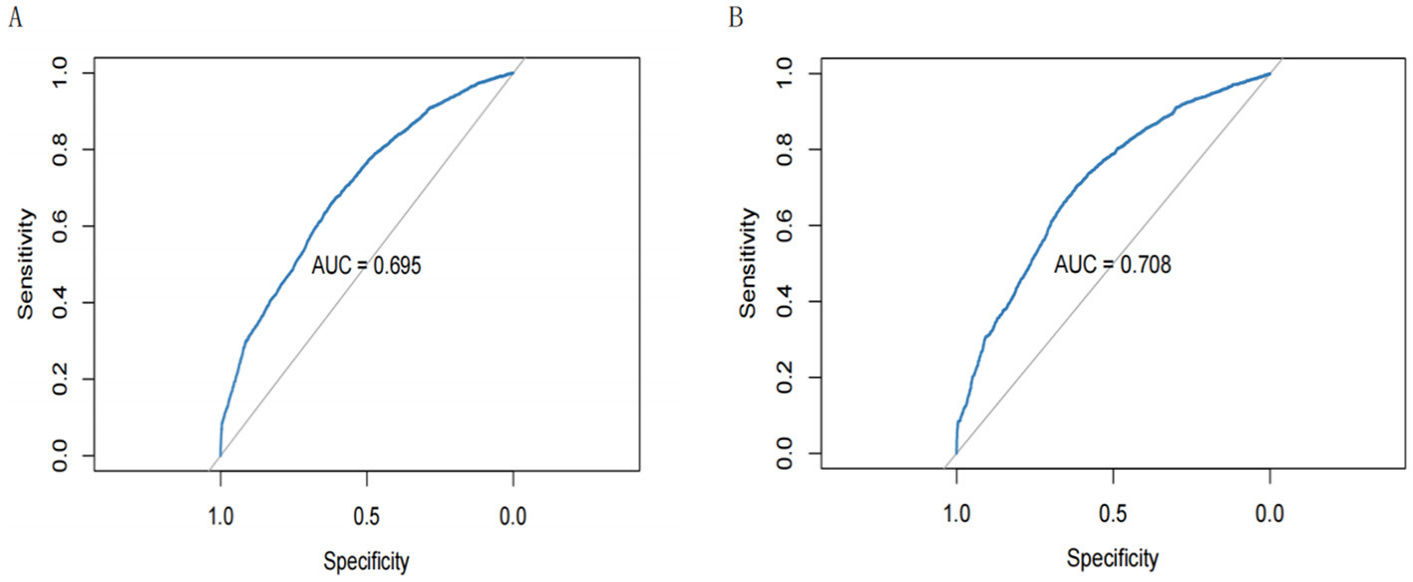
**(A)** Receiver operating characteristic (ROC) curves for the training set; **(B)** ROC curves for the test set.

In this study, the predictive performance of the prediction model constructed based on the training set was evaluated by the area under the subject curve (AUC). As shown in Figure 8, the AUC of the prediction model in the training set is 0.695 (95% CI 0.6844–0.7056), with a *p*-value of < 0.05, whereas the AUC of the prediction model in the test set is 0.708 (95% CI 0.6928–0.7225), with a *p*-value of < 0.05. The above results indicate that our prediction model performs significantly with moderate discriminative ability, reflecting good predictive performance.

## 4 Discussion

In this investigation, we developed and validated an arthritis prediction model based on ENs and BNs, and our results revealed factors associated with the development of arthritis, showing that several factors are directly or indirectly linked to arthritis. First, depression is a strong risk factor directly associated with arthritis and promotes its development. Second, we found that age is a direct correlate of the incidence of arthritis in the older adults. In addition, gender was also found to be an indirectly related factor.

In recent years, BNs have gained great attention as a data-driven model in clinical research. On the basis that the inclusion of a larger number of variables might lead to a more complex network, we have developed and validated an arthritis prediction model based on ENs and BNs, which can be used to assess the risk of arthritis in middle-aged and older adults in the Chinese community, and which incorporates 12 predictive factors such as depressive symptoms, BMI, gender, hypertension, diabetes mellitus, joint pain and smoking. Previous studies have typically explored disease risk factors based on logistic regression, which relies on OR values to determine the magnitude of association strength, but which does not provide a comprehensive overview of the overall association between risk factors and thus cannot detect direct or indirect risk factors (10). In contrast, BNs have the advantage of accurately detecting the risk factors for a particular disease, vividly depicting the complex network mechanisms between a disease and its risk factors and determining the correlation between these risk factors (16).

The results of this study reveal factors associated with the development of arthritis, showing that several factors are directly or indirectly linked to arthritis. First, depression is a close risk factor directly associated with arthritis and promotes its development. Inflammation is one of the characteristics of depressive symptoms, and elevated levels of pro-inflammatory factors can be observed in the absence of autoimmune diseases (17). Increased levels of depression-related pro-inflammatory cytokines (such as TNF-α, IL-6, and IL-1) can activate the hypothalamic-pituitary-adrenal (HPA) axis and the sympathetic nervous system via various pathways. This activation may result in immune system dysfunction, thereby forming a significant pathophysiological connection between depression and chronic conditions such as arthritis. Central inflammation in depression induces a systemic inflammatory response and might contribute to the development of autoimmune diseases such as arthritis. In addition, some epidemiological evidence suggests that depression is a risk factor for rheumatoid arthritis. A cohort based on the National Health Insurance Research Database of Taiwan has shown that the incidence of depression is significantly higher in patients with rheumatoid arthritis than in the non-rheumatoid arthritis population [15.69 vs. 8.95 per 1,000 person-years (PYs)], and at the same time, the incidence of rheumatoid arthritis is significantly higher in the depressed population than in the non-depressed population (2.07 vs. 1.21 per 1,000 PYs) (18), i.e., the study demonstrates a strong bidirectionality between depression and rheumatoid arthritis. Meanwhile, another retrospective cohort study has revealed that patients who have major depression and who use antidepressants have a lower risk of rheumatoid arthritis. The effect of age on arthritis and the role of age as an important factor in the development of arthritis have been widely recognized (19).

The prevalence of arthritis increases with age. Aging is an inevitable problem for the human body, and age-related chronic diseases are a major contributor to global disease incidence and mortality. Epidemiological studies have identified a wide range of chronic diseases as being age-related, including atherosclerosis, osteoarthritis, rheumatoid arthritis, and cancer. One investigation has suggested that age-related self-DNA accumulation is an important reason for the increased incidence of rheumatoid arthritis in middle-aged and older patients (20). Moreover, older people are more susceptible to various changes in their lifestyle and the environment, all of which might increase the risk of developing arthritis and can lead to arthritis flare-ups. In addition, age might directly affect various body systems in middle-aged and older adults, increasing their susceptibility to arthritis. Our findings have also identified age as a direct correlate affecting the incidence of arthritis in the older adults. This finding therefore highlights the need for the age-specific testing of middle-aged and older adults and the significance of tailoring interventions for this population.

With respect to gender, one study has shown that the incidence of rheumatoid arthritis is three times higher in women than in men (21). The occurrence of arthritis in middle-aged and older adults is influenced by gender, as evidenced by previous epidemiological studies (22). A Mendelian randomization study has also demonstrated a positive causal relationship between sex-hormone-binding globulin (SHBG) and osteoarthritis (23).

The strength of our study lies in the effective identification of factors associated with arthritis in middle-aged and older adults in the Chinese community by using BNs, revealing the complex network of relationships between them and arthritis. Although traditional logistic regression can identify risk factors for arthritis, it fails to elucidate the specific role of these risk factors in the development of arthritis. However, our study has some limitations. The first is that the directed edges of BNs do not represent causal relationships, but only conditional reliance between nodes. The second is that missing data are inevitable in large public databases, and although the missing values of some samples can be filled in based on some statistical methods, a certain selection bias is still introduced. Finally, future comprehensive explorations of arthritis should include other variables, in addition to those included in this study, in order fully to explore the correlation between arthritis and these factors.

Based on BNs, we have found that age and depressive symptoms are direct correlates affecting the incidence of arthritis in the older adults, whereas gender exhibits an indirect correlation. BNs can thus effectively reveal the complex network of relationships between disease and its correlates. Moreover, we have developed and validated a prediction model for the occurrence of arthritis in middle-aged and older adults in the Chinese community based on BNs. The prediction model has good sensitivity, specificity, and discriminatory properties. This finding can be used as a reference for public health departments possibly enabling them to reduce the occurrence of arthritis in middle-aged and older adults. The prediction model will also help community health organizations and clinicians to screen and predict the incidence of arthritis in the community and to implement early intervention measures.

## Data Availability

The raw data supporting the conclusions of this article will be made available by the authors, without undue reservation.
